# Contact dermatitis of the lips due to late-type sensitization against dalbergiones in a wooden recorder 

**DOI:** 10.5414/ALX01323E

**Published:** 2018-09-01

**Authors:** C. Pföhler, W. Tilgen

**Affiliations:** Klinik für Dermatologie, Venerologie und Allergologie, Universitätsklinikum des Saarlandes, Homburg/Saar

**Keywords:** dalbergiones, recorder, contact dermatitis, (S)-4’-hydroxy-4-metho- xydalbergione, (S)-4-methoxydalbergione

## Abstract

Background: Late-type sensitizations against wood are rare and are mostly seen in patients with occupational contact with saw dust. Generally, commercial wooden products only lead to sensitization or dermatitis in cases when contact to the unprotected skin is direct, intensive and of longer duration, i.e. by wearing wooden jewelry or by playing wooden instruments. Causative for the sensitization process are ingredients of the wood core such as alkaloids, glycosides, anthrachinones, saponines, phenols, catechols, flavonoids, cumarins, and benzo-, naphtho-, furano-, and phenanthrenquinones and their precursors. Case report: We report the case of a 70-year-old patient who developed cheilitis after playing different wooden recorders made of African blackwood, rosewood, cedar, olive, and pear. Methods: Patch testing with baseline series of contact allergens and saw dust of the recorders were performed as well as skin prick tests with common inhalant allergens and saw dust of the recorders. Results: Patch testing showed sensitizations against African blackwood, rosewood, fragrance mix 2 and hydroxyisohexyl-3-cyclohexene carboxaldehyde. Skin prick testing was without pathological results. Conclusion: African blackwood and rosewood contain (S)-4’-hydroxy-4-methoxydalbergione and (S)-4-methoxydalbergione. As a result of the chemical affinity between the dalbergiones, cross-reactions between different woods are observed. The case presented shall show the diagnostic procedure in cases in which sensitizations against wood components are suspected.

**German version published in Allergologie, Vol. 33, No. 9/2010, pp. 410-412**

## Introduction 

Dalbergia melanoxylon is also known as African Blackwood and native to Africa, mainly Tanzania and Mozambique. The dense heart wood is of a purple-blackish color with black stripes and can be mistaken for ebony. Due to its nice color and to its hardness it is frequently used for wooden instruments like clarinets, oboes and recorders, but also for chess pieces and combs [2, 4]. 

## Case report 

**Figure 1.**
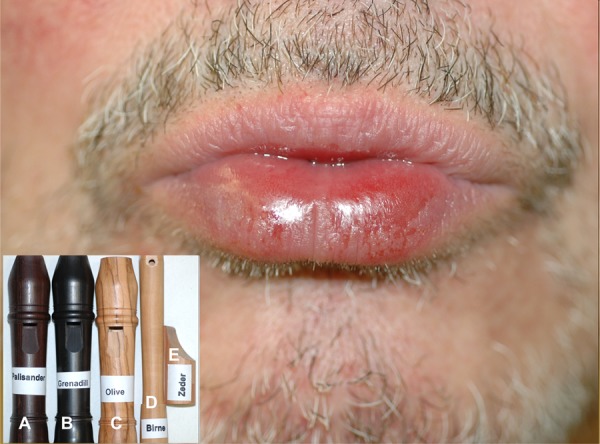
Erosive cheilitis 24 hours after playing a recorder made of African blackwood. Patient’s recorders (inset): A: rosewood; B: African blackwood; C: olive; D: pear; E: cedar.

A 70-year-old patient presented to our allergology department with a 2-year history of relapsing erosive cheilitis (Figure 1). The changes in the skin had always been treated with low-potency topical corticosteroids which had always resulted in a remission within several days. The specific patient history revealed that the patient had been playing different types of recorders with mouthpieces made of African blackwood, rosewood, cedar, olive or pear in his leisure time (Figure 1, inset). He had already suspected an association between his hobby and the episodes of cheilitis, but was not sure which of the mouthpieces had caused the lesions. When using a plastic mouthpiece the condition did not occur. 

## Methods 

We carried out patch tests using the standard allergens (Almirall Hermal, Reinbek, Germany) and saw dust from the mouthpieces of his recorders (10% in petrolatum). The allergens were applied on his upper back using Finn Chambers^®^ on Scanpor (Epitest, Tuusula, Finland) and attached with Fixomull Stretch Tape (BSN Medical, Hamburg, Germany). The allergens were removed 24 hours later to avoid irritant reactions. The test results were checked after 24, 48 and 72 hours according to the guidelines published by the International Dermatitis Research Group (ICDRG).

Inhalable allergens (grass pollen mix, tree pollen mix, mugwort, Dermatophagoides pteronyssinus, Dermatophagoides farinae and feathers) were tested in a routine prick test using commercially available test solutions (Bencard, Munich, Germany). Furthermore a skin prick test with saw dust from the different mouthpieces of his recorders (1% in water) was carried out. The results of this skin prick test were checked after 20 minutes and after 24 hours. The patch testing and the prick testing were also carried out in 7 control persons who had presented to our hospital for diagnostic work-up of occupation-related sensitizations. These control persons were informed about the aim of the testing and about the possibility of an active sensitization and gave their written consent to the additional testing.

## Results 

**Figure 2.**
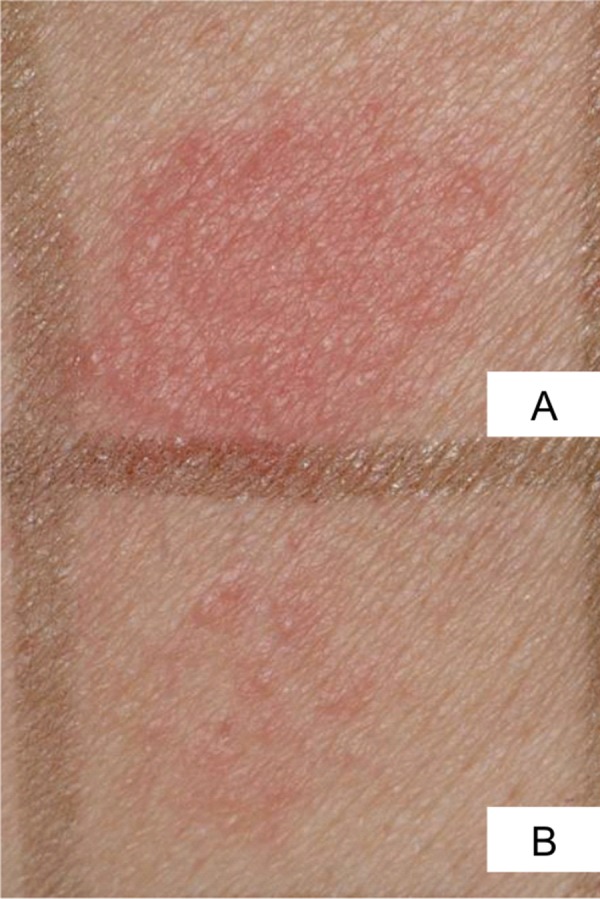
Positive patch test reactions to African blackwood (A) and rosewood (B) after 48 hours.

The patch testing showed positive reactions to African blackwood and rosewood (Figure 2) as well as to fragrance mix 2 and hydroxymethylpentylcyclohexenecarboxaldehyde (Lyral). The sensitization to the fragrance mix was clinically irrelevant according to the patient history. Skin prick testing was without pathological results. The tested control persons did not show any abnormalities in the patch and prick tests. 

## Discussion 

Dalbergia melanoxylon or African blackwood is a tropical hardwood that can easily be mistaken for ebony (Diospyros species) due to its color. The heart wood is very hard and dense and is used for the production of wooden recorders due to its nice color as well as its temperature and moisture resistance. All Dalbergia species can induce irritant or allergic reactions in skin and mucosa [6]. Irritant or contact allergic skin lesions or asthma-like symptoms are usually only observed in persons with occupational contact to tropical woods and their saw dusts (sawmills, wood-working industry). Only very rarely they arise due to contact with wooden instruments, like in the case of the so-called “fiddler’s neck”, or due to skin contact with jewelry like bracelets or necklaces made of tropical wood [1, 3, 5]. 

The reason for allergic reactions after contact with African blackwood are, like in the case of other Dalbergia species, the quinones (S)-4’-hydroxy-4-methoxydalbergione and (S)-4-methoxydalbergion. As a result of the structural similarity of all dalbergiones cross-reactions between different woods are observed [7]. This was also the case in our patient who had positive patch test reactions to African blackwood and rosewood. 

The case presented here is supposed to remind dermatologists of the fact that, in rare cases, wooden instruments can cause contact allergic skin reactions. When sensitization to certain woods is suspected, patch and prick tests with alcohol or water extracts of the suspected woods should be carried out [2, 5]. As these extracts are usually not commercially available, patch testing can be carried out with an individually produced test formulation (10% saw dust in petrolatum). It has to be noted that for several woods, like teak, this concentration might be too high and could cause irritant reactions or active sensitizations [2]. In such cases a titration series should be used. 

## Conflict of interest 

None.
